# Living donor liver transplantation for Budd–Chiari syndrome: Overcoming a troublesome situation

**DOI:** 10.1097/MD.0000000000005136

**Published:** 2016-10-28

**Authors:** Cengiz Ara, Sami Akbulut, Volkan Ince, Serdar Karakas, Adil Baskiran, Sezai Yilmaz

**Affiliations:** Department of Surgery and Liver Transplant Institute, Inonu University Faculty of Medicine, Malatya, Turkey.

**Keywords:** anastomosis technique, Budd–Chiari syndrome, liver transplantation, living donor liver transplantation, technical difficulties

## Abstract

**Background:**

The aim of the study was to report the detailed surgical techniques of living donor liver transplantation (LDLT) in patients with Budd–Chiari syndrome (BCS).

**Methods:**

Demographic and surgical techniques characteristics of 39 patients with BCS who underwent LDLT were retrospectively reviewed. Thirty-two of them had native vena cava inferior (VCI) preservation and 6 had retrohepatic VCI resection with venous continuity established by cryopreserved VCI (n = 4) or aortic graft (n = 2). In 1 patient, the anastomosis was established between the graft hepatic vein (HV) and the suprahepatic VCI. For preservation of the native VCI, immediately before the graft implantation, the thickened anterior, and right/left lateral walls of the recipient VCI were resected caudally and cranially until the intact vein wall was reached, and then an anastomosis was created between the (HV) of the graft reconstructed as a circumferential fence and the reconstructed recipient VCI. For resection of the retrohepatic VCI, the anastomosis was created with the same technique in all 6 patients in whom VCI was reformed by using a vascular graft.

**Results:**

Post-LT complications developed in 19 of the patients. Complications related to the biliary anastomosis accounted for 12 of these cases, with 11 treated by PTC and/or ERCP, and 1 by hepaticojejunostomy. Two of the 39 patients developed recurrent BCS and were treated by interventional radiological methods. Thirteen patients died and none were related to the BCS recurrence.

**Conclusion:**

Favorable outcomes are achievable with LDLT treatment of patients with BCS, which carries important implications for countries with inadequate cadaveric donor pools.

## 1 Introduction

Budd–Chiari syndrome (BCS) was first described by Budd in 1845 and then expanded by Chiari in 1899. The long-standing clinical definition of this life-threatening condition is obstruction of hepatic venous drainage due to various causes that leads to progressive hepatic congestion, liver failure, and portal hypertension; the obstruction may occur at the level of the hepatic venules, the large hepatic veins, the vena cava inferior (VCI), or the right atrium.^[^1–4^]^ In current medical practice, BCS is classified as either primary or secondary, with the former being attributable to intrinsic intraluminal thrombosis or the development of venous webs and the latter being attributable to intraluminal invasion by malignant tumor or extraluminal compression by solid tumor, or by a cystic lesion, such as associated with echinococcal infection.^[^1 4^]^ Furthermore, BCS cases are categorized according to the obstruction location, with involvement of either small hepatic veins, main hepatic veins or the VCI exclusively, or a combination of the main hepatic veins and VCI.^[1]^

Several invasive and noninvasive therapeutic modalities have been developed and are currently in clinical use as treatment of BCS. These modalities include anticoagulation therapy, thrombolytic therapy, diuretic drug therapy, percutaneous transluminal angioplasty with and without stenting, transjugular intrahepatic portosystemic shunting, surgical shunting, and liver transplantation (LT).^[1]^ The indications for LT in the BCS patient population include fulminant hepatic failure, cirrhosis, and failure of a previous treatment attempt with portosystemic shunting or nonsurgical therapeutic modalities.^[^1 3^]^ Most of the available literature on LT for BCS reports data on deceased donor liver transplantation (DDLT), and only a few reports have addressed applications and outcomes of living donor liver transplantation (LDLT).^[2]^

In BCS patients undergoing LDLT, hepatic venous reconstruction can be more difficult than in the DDLT procedure, particularly in construction of adequate anastomoses.^[2]^ In DDLT the recipient's VCI is generally replaced by the donor's, since the liver graft contains the retrohepatic VCI.^[^2 3^]^ In contrast, in LDLT the recipient's VCI is preserved, most frequently via the piggy-back technique. In the absence of a donor retrohepatic VCI, the anastomosis between the recipient's hepatic vein cuff and the graft's hepatic vein is created using a partial liver graft.^[^2 3^]^ Specific venous drainage models are required which correspond to the level and size of the stenotic or the obliterated retrohepatic VCI.^[^2 3^]^

### 1.1 Primary and secondary outcomes

The primary objective of this study was to report the details of the surgical techniques used in LDLT of 39 patients with BCS at our liver transplant institute. The secondary objective was to discuss the clinical implications and the success rate of these surgical techniques in comparison to those reported by prior studies in the literature.

## 2 Materials and methods

### 2.1 Study design

Between 8 March 2002 and 26 November 2015, 1615 LTs [LDLT: 1278(79.1%); DDLT: 337(20.9%)] were performed at the Inonu University Faculty of Medicine's Liver Transplantation Institute (Malatya, Turkey). Of these total LTs, 43 (2.66%) were undertaken to treat signs and symptoms of BCS, with 39 patients undergoing LDLT and 4 undergoing DDLT. The 4 DDLT cases were excluded from this study to keep with the primary goal of presenting the details of the hepatic outflow reconstruction techniques used in cases undergoing LDLT for BCS (flowchart). Local ethics committee approval was not received because of this study is a retrospective clinical study.

**Figure:**
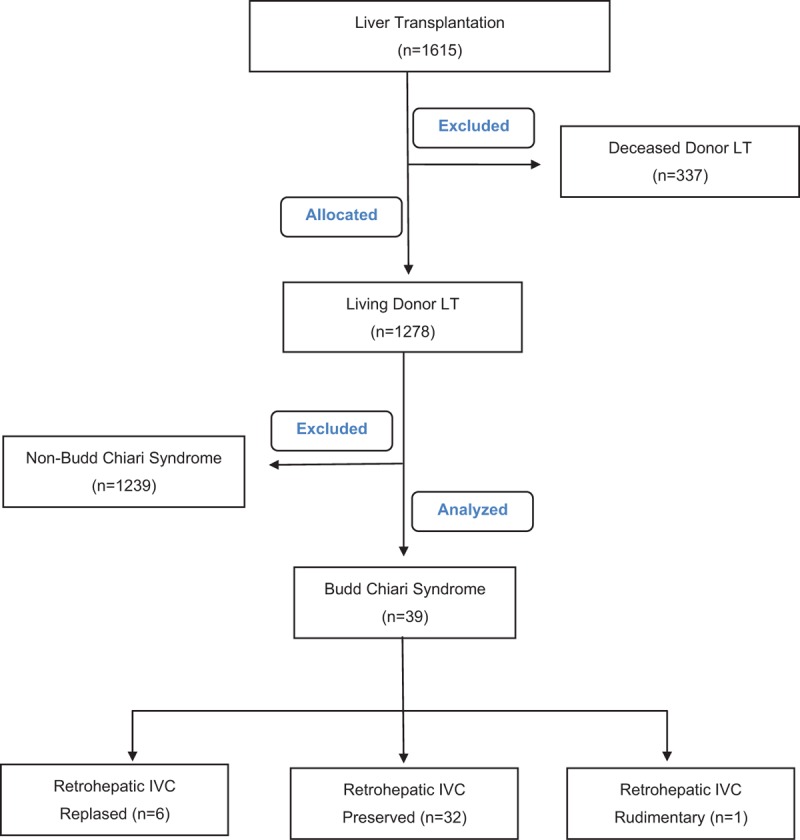


All BCS cases had been diagnosed on the basis of 1 or more features of BCS evidenced upon clinical presentation, physical examination, radiological testing, intraoperative observation, and histopathological analysis. Patients showing thrombus, fibrosis, or a web in hepatic veins or VCI during recipient hepatectomy were deemed to have BCS. Patients with evidence of complete or incomplete thrombus in hepatic veins and/or VCI in radiological examination (Doppler ultrasonography and/or multidetector computed tomography) were diagnosed with BCS, irrespective of underlying causes. Depending on the possible etiologies indicated by biochemical and radiological testing, the BCS was classified as primary (prothrombotic disorder, factor V Leiden mutation, paroxysmal nocturnal hemoglobinuria, etc.) or secondary (associated with hepatocellular carcinoma, non-parasitic cyst, echinococcal disease, epithelioid hemangioendothelioma, etc.).^[^1 2^]^ The BCS cases for which the examinations provided no indication of the underlying pathology were categorized as idiopathic. For our study, the BCS cases were grouped according to acute and chronic BCS status, according to the timing of appearance of the various characteristic clinical signs and symptoms. Acute BCS was characterized by development of coagulation disorders, resistant ascites, liver enlargement, and elevated markers on liver function tests, within the last month.^[2]^ Chronic BCS was characterized by portal hypertension and relatively less elevated markers on liver function tests, over 1 month previous.^[2]^

All LDLT-treated BCS patients received a heparin infusion course after the transplantation. Sufficiency of the heparin therapy was monitorized via assessment of activated partial thromboplastin time (aPTT; normal value: 30–40 seconds, target value: 60–90 seconds). At the end of 1 week, the heparin therapy was switched to coumarin therapy to achieve a stable level of the international normalized ratio (INR; approaching 2); in addition, a dosing regimen of 100 mg/day acetylsalicylic acid was commenced at this time. At the end of month 6 post-LDLT, the coumarin therapy was stopped, but the acetylsalicylic acid therapy was continued at the same dosage. Immunosuppressive therapy was administered in a patient-tailored manner, according to underlying disorders, renal function and hematological parameters.

### 2.2 Surgical models for recipients with preservation of VCI

To maximize the benefit of liver grafts obtained from living donors, a venous drainage model with a wide orifice was created on the backtable. For right lobe grafts, all hepatic veins with a diameter of ≥5 mm (V5, V8, and inferior right hepatic vein [IRHV]) were extended toward the right hepatic vein orifice by using artificial or homologous vascular grafts. In this so-called all-in-one drainage model, a circumferential fence was formed with the saphenous vein graft, thereby ensuring that it includes all venous orifices. The diameter of the right lobe venous drainage model that is formed with this technique is usually ≥5 cm. In the limited number of cases in which the IRHV was > 5 cm away from the hepatic vein, the IRHV was anastomosed directly to the VCI. For left lobe grafts and left lobe lateral segmental grafts, a circumferential fence with a diameter of 3 to 5 cm was formed by wrapping the saphenous vein graft around the left haptic vein.

**Figure 1:**
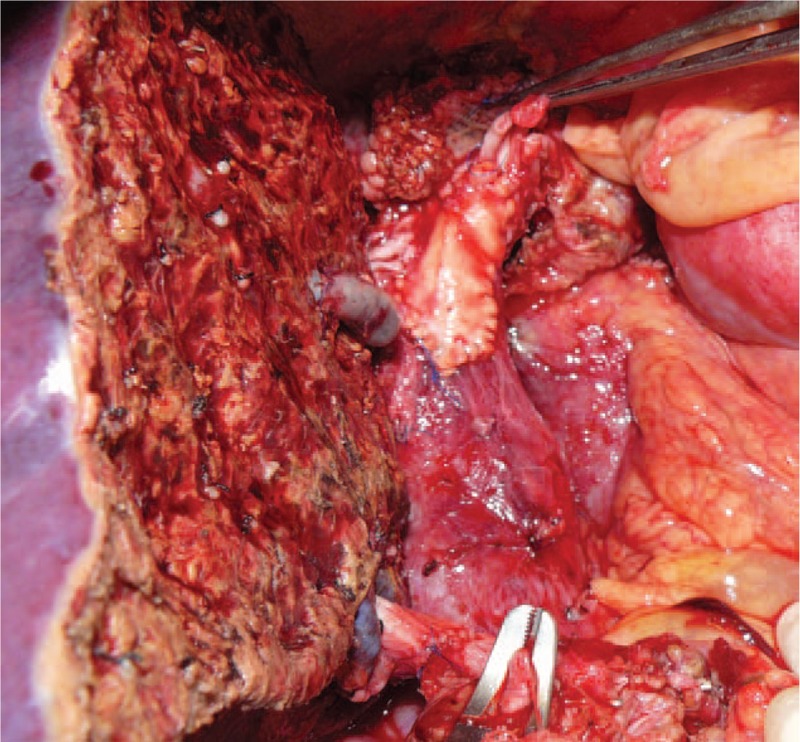
Intraoperative image of a patient who underwent aortic patch plasty to the anterior surface of the VCI in order to relieve stenosis. VCI = vena cava inferior.

Native VCI was preserved in 32 of the 39 total patients who underwent LDLT for BCS. After explanting the native liver, the fibrous tissues around the VCI were dissected using scissors or an ultrasonic dissector. The dissection procedure was extended cranially, until the border of the intact suprahepatic VCI was reached and in most cases was continued until proximal to the diaphragmatic crus; the dissection was also extended caudally in a similar manner until the suprarenal VCI was reached. The veno-venous bypass procedure was not used in any patient. After completion of the dissection procedure, cross-clamps were applied to the suprahepatic and suprarenal VCI, taking into account the borders of the intact VCI. In cases that were to undergo right lobe implantation, the VCI's thickened anterior and right lateral walls were excised in a manner that facilitated inclusion of the orifice of the right hepatic vein; for the left lobe grafts, the VCI's thickened anterior and left lateral walls were excised in a manner that facilitated inclusion of the orifice of the left hepatic vein. The excision procedure involving the thickened vein wall was continued cranially and caudally, until intact vein wall was reached. Hence, a large orifice was formed on the native VCI. The diameter of the venous drainage model constructed on the liver graft on the backtable was adjusted to match the new orifice on the recipient's VCI. A vascular anastomosis was created with the eversion technique, using a 5/0 prolene suture. Other stages of the implantation were performed by the standard techniques. VCI stenosis developed after the anastomosis in 1 of the 32 patients in who this technique was used; in that patient, patch plasty to the VCI was performed using an aortic graft material and no post-procedural complications occurred (Fig. 1).

### 2.3 Surgical models for recipients with reconstruction of the VCI

**Figure 2:**
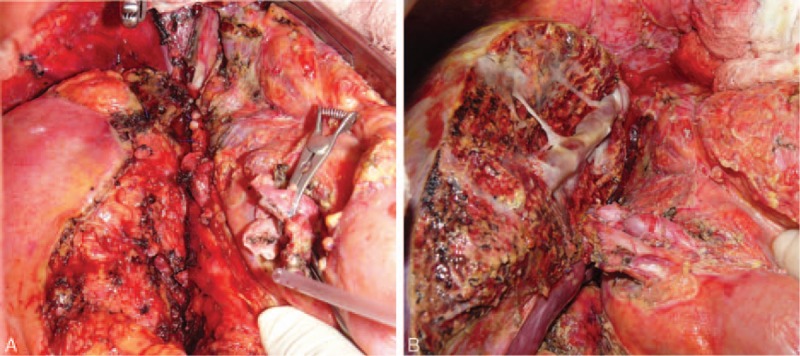
Postexcision image of the rudimentary retrohepatic VCI. The clamp is holding the suprahepatic VCI (A). The liver graft, in which a venous drainage model was formed with the all-in-one technique, was directly anastomosed to the suprahepatic VCI (B). VCI = vena cava inferior.

**Figure 3:**
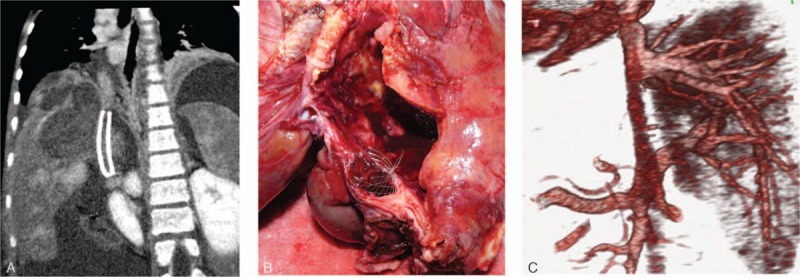
Coronal CT image obtained after placement of a stent to the VCI by an interventional radiological method in order to relieve BCS (A); severe venous injury secondary to stenting is shown in the explanted retrohepatic VCI of the liver (B). CT image showing venous drainage after the LDLT (C). BCS = Budd–Chiari Syndrome, CT = computed tomography, LDLT = living donor liver transplantation, VCI = vena cava inferior.

**Figure 4:**
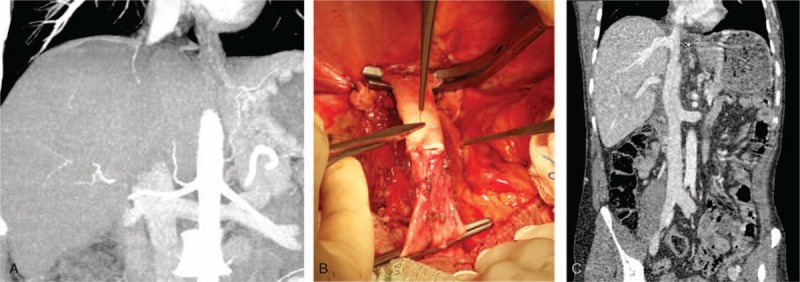
Coronal CT image showing complete occlusion of a retrohepatic VCI (A). After the occluded segment of the VCI was excised, venous continuity was established with an aortic vascular graft (B). Coronal CT image showing the posttransplant venous drainage (C). CT = computed tomography, VCI = vena cava inferior.

**Figure 5:**
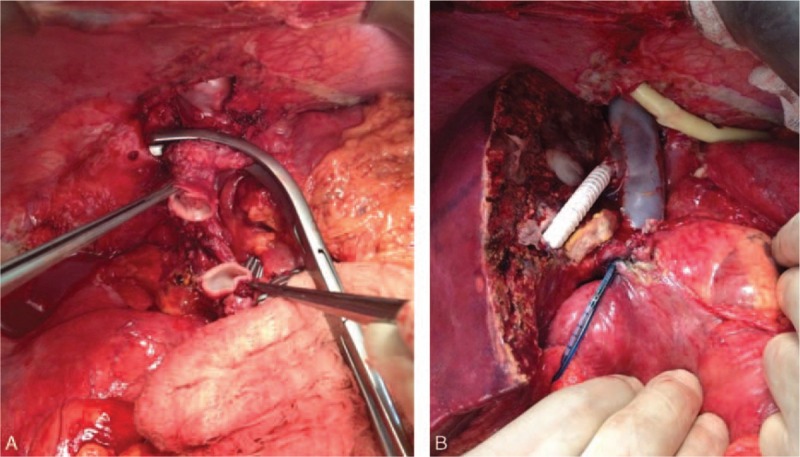
Views from the operation field. Resected state of the occluded retrohepatic segment of the VCI. A vascular clamp was applied just distal to the right atrium (A). The VCI is shown after replacement with a vascular graft (B). VCI = vena cava inferior.

For 7 of the 39 patients, the native VCI could not be utilized for various reasons (with brief summaries of these cases provided forthwith). A 21-year-old male patient with idiopathic BCS underwent LDLT. It was observed during the exploratory stage of the surgery that the VCI was in the form of a fibrous band; however, the suprahepatic VCI was suitable for anastomosis and an anastomosis was created between the suprahepatic VCI and the right lobe liver graft (Fig. 2A and B). A 32-year-old female patient with BCS secondary to cystic echinococcosis underwent LDLT. It was observed during the exploratory stage of the surgery that the VCI was rudimentary; a cryopreserved VCI graft was placed between the suprahepatic and suprarenal VCI segments after native VCI resection, after which an anastomosis was created between the liver graft prepared on the backtable and the newly formed VCI. Four months later, that patient developed severe pancreatitis after an endoscopic retrograde cholangiopancreatography (ERCP) procedure performed for a biliary stricture and died despite aggressive medical treatment. A 12-year-old female patient with BCS secondary to cystic echinococcosis underwent LDLT after first being placed with a stent to the VCI, which had produced no change in the clinical signs of BCS. It was observed during the exploratory stage of the surgery that the stent had caused severe intimal injury in the VCI. Thus, after applying cross-clamps to the suprahepatic and suprarenal VCI, the injured VCI segment was resected and venous continuity was established by a cryopreserved cadaveric aorta graft. Then, an anastomosis was constructed between the lateral segment of the left lobe that had been obtained from a living donor and the newly formed VCI (Fig. 3A–C). During follow-up the patient developed chronic rejection of the graft and a DDLT was performed; however, the patient died due to postoperative severe sepsis.^[5]^ A 29-year-old male patient with idiopathic BCS underwent LDLT. It was observed during the exploratory stage of the surgery that a long segment of the VCI was both occluded and fibrotic; therefore, after application of the cross-clamps, the native liver was totally resected together with VCI. After venous continuity was established with a cryopreserved cadaveric aortic graft, an anastomosis was created between the right lobe liver graft and the newly formed VCI (Fig. 4A–C).^[6]^ A 15-year-old male patient with idiopathic BCS underwent LDLT. After explanting the native liver, the retrohepatic VCI was found to be thrombosed and fibrotic, and during dissection it was observed that the occluded segment extended to the right atrium. Hence, the right diaphragm was opened and a cross-clamp was applied at the level of the right atrium. A distal cross-clamp was then applied to the suprarenal VCI. After explanting the fibrotic native VCI in between, venous continuity was established with a cryopreserved cadaveric VCI (Fig. 5A and B) and an anastomosis was created between the right lobe liver graft and the newly formed VCI.^[7]^ A 28-year-old woman with BCS secondary to alveolar echinococcosis underwent LDLT. The dissection was quite difficult, owing to both diffuse perihepatic fibrosis and alveolar disease involving the VCI. A significant portion of the VCI was resected after total vascular clamping. The defect that had developed in the native VCI was not suitable for reconstruction and thus the retrohepatic VCI was resected and a cadaveric VCI was used for establishing venous continuity. Afterwards, an anastomosis was created between the right lobe liver graft and the newly formed VCI; however, the patient developed hepatic artery thrombosis the next day and died.^[8]^ A 33-year old woman with idiopathic BCS underwent LDLT. The retrohepatic VCI was found to be thrombosed and severely fibrotic; resection was carried out, owing to its unsuitable nature for anastomosis, and venous continuity was established with a cryopreserved cadaveric VCI graft. An anastomosis was then created between the right lobe liver graft and the newly formed VCI. The patient developed postoperative hepatic artery thrombosis and underwent DDLT, after which the hepatic artery thrombosis recurred and the patient died.

## 3 Results

### 3.1 Demographic and clinical characteristics

**Figure 6:**
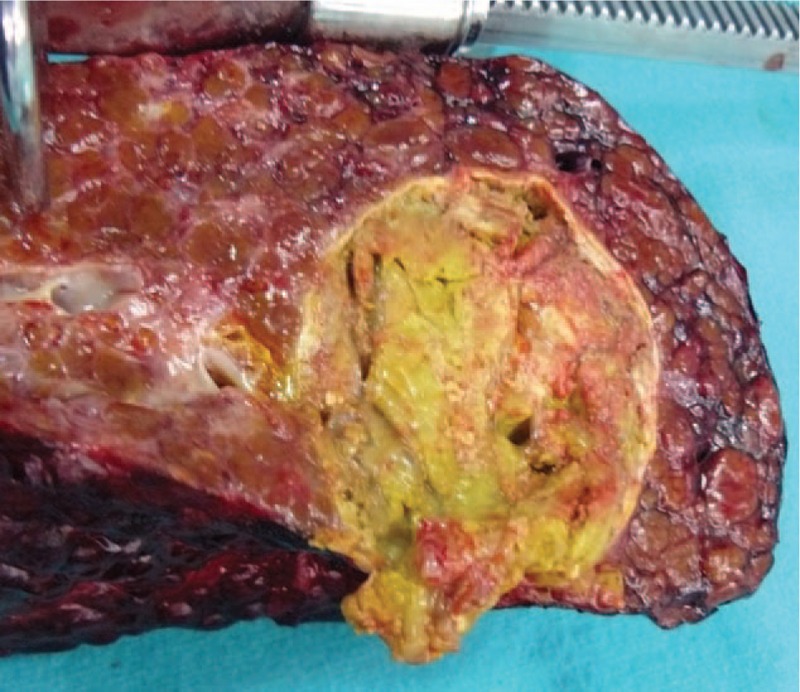
A section of liver explanted from a patient with hemangioendothelioma.

This study included a total of 39 patients (18 males and 21 females) with BCS, ranging in age from 2 to 58 years old (Mean ± SD: 28.6 ± 12.5 years). For 31 of the patients, no underlying cause of the BCS was determined; for the remaining, 8 patients the primary underlying causes were cystic echinococcosis (n = 2), HCC (n = 2), alveolar echinococcosis (n = 1), myeloproliferative disorder (n = 1), hemangioendothelioma (n = 1) (Fig. 6), and paroxysmal nocturnal hemoglobinuria (n = 1). According to these findings, 31 patients had idiopathic BCS, 6 had secondary BCS and 2 had primary BCS. Thirty-five of the total 39 patients underwent LDLT for liver disease on the background of chronic BCS, whereas the remaining 4 underwent LDLT for acute liver failure that developed upon the basis of acute BCS. The Child status of the disease was B in 22 patients, C in 13, and the remaining patients had Child A disease; the mean ± SD Child score was 8.7 ± 2.0 (range: 5–13) and the mean ± SD score of model for end-stage liver disease (commonly known as the MELD score) was 15.5 ± 7.4 (range: 6–39). The means ± SD of graft weight, cold ischemia time, and total operative time were 698 ± 219 g (range: 213–1025 g), 193 ± 104 minutes (42–500 minutes), and 540 ± 190 minutes (300–1160 minutes), respectively. Twenty-five of the patients were administered 1 to 9 units of erythrocyte transfusion during the surgery, whereas 11 patients did not receive any erythrocyte transfusion; no information of transfusion was obtained for the remaining 3 patients. Seventeen patients were administered 1 to 8 units of fresh frozen plasma during the surgery, whereas 16 patients did not receive any fresh frozen plasma transfusion; no information of fresh frozen plasma transfusion was obtained for the remaining 3 patients. Only 7 of the total 39 patients received autologous blood transfusion via the Cell Saver device to address bleeding episodes during the dissection. Twenty-nine patients were implanted with a right lobe liver graft, 5 patients were implanted with a left lobe liver graft, and 5 patients were implanted with a left lobe lateral segment of liver.

### 3.2 Postoperative complications

Nineteen of the total 39 (48.7%) patients developed postoperative complications. Among the 12 patients who developed a biliary complication, biliary stricture was detected in 7 and biliary leak was detected in 5. Eleven of these biliary-complication patients were treated by percutaneous transhepatic cholangiography and/or endoscopic retrograde cholangiopancreatography, in several sessions; the remaining 1 patient necessitated a hepatico-jejunostomy operation. One of the patients treated by ERCP developed severe pancreatitis post-treatment and died. Two of the 39 (5.1%) patients experienced BCS recurrence, which arose as a complication related to hepatic venous outflow during postoperative follow-up. A 15-year-old patient developed severe stenosis and thrombus at the junction of the VCI and hepatic vein, as detected by vena cavography, at 19 months post-transplantation; a successful balloon dilatation, with a 12 × 40 balloon, was performed in the stenotic segment. A 30-year-old patient developed a severe stricture in the VCI at 6 months, and 3 separate balloon dilatation procedures were performed, with the final session including placement of a 20 × 50 mm stent in the occluded segment; and several more balloon dilatations were carried out over the stent. As of the writing of this paper, these 2 patients have attended regular follow-up and shown no indications of complications related to venous outflow. Finally, 3 patients received repeat laparatomy after bleeding was detected from drains, occurring in the early postoperative period; hemostasis was achieved and no episodes of bleeding recurrence occurred. Other complications were summarized in Table 1 .

**Table 1:**
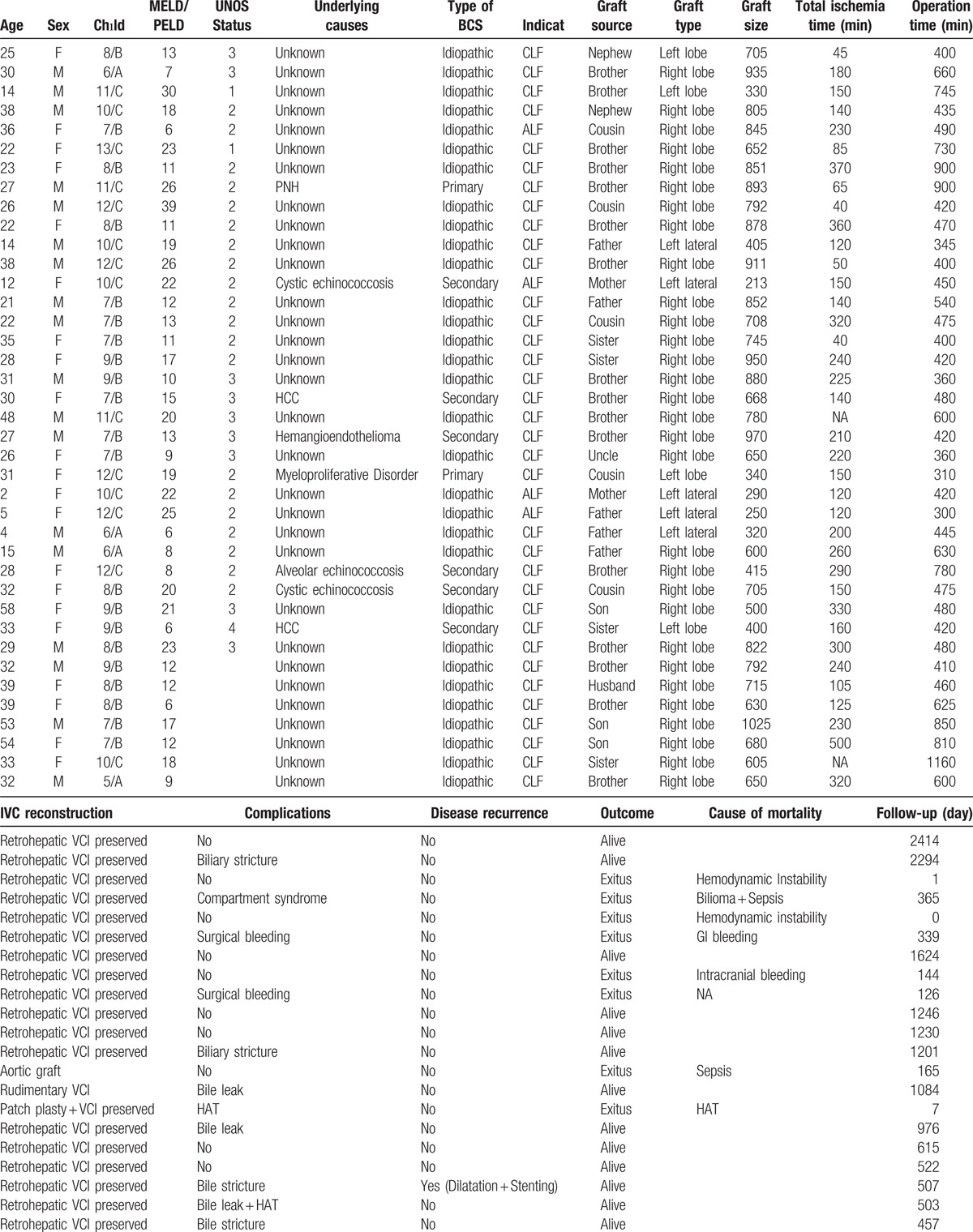
Demographic and clinical characteristics of the 39 patients with BCS who underwent LDLT included in this study.

**Table 1 (Continued):**
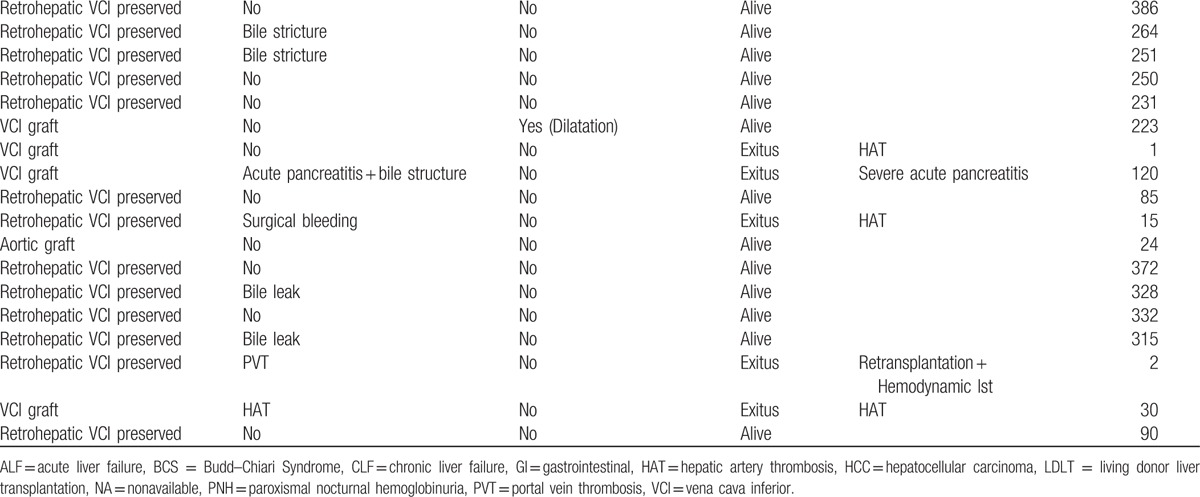
Demographic and clinical characteristics of the 39 patients with BCS who underwent LDLT included in this study.

### 3.3 Follow-up

Thirteen of the total 39 patients died, for a mortality rate of 33.3%. The mean ± SD of days to death post-LDLT was 491 ± 590 (range: 0–2414 days). None of the deaths occurred secondary to complications related to hepatic venous outflow or BCS recurrence. The deaths were determined to be due to hepatic artery thrombosis (n = 4), hemodynamic instability (n = 2), sepsis (n = 2), severe acute pancreatitis plus hemodynamic instability (n = 1), gastrointestinal bleeding (n = 1), portal vein thrombosis plus hemodynamic instability (n = 1), and intracranial bleeding (n = 1); 1 death had no clear reason associated with it. The detailed demographic and clinical data of the study population are summarized in Table 1 .

## 4 Discussion

Although many treatment modalities are currently available for BCS, the definitive treatment option for this condition is LT, and 10% to 20% of patients undergo DDLT or LDLT.^[^1–3^]^ The first DDLT in a BCS patient was performed by Starzl et al^[9]^ in 1974 and involved a 22-year-old female patient with polycythemia vera. Since then, >1000 patients with BCS have undergone DDLT. According to the results of 2 studies analyzing 1080 patients with BCS who had undergone DDLT between 1976 and 2009, the survival rates at 1, 3, 5, and 10 years after DDLT were 64% to 95%, 45% to 95%, 45% to 95%, and 65% to 84%, respectively.^[^2 10 11^]^ The first LDLT was performed by Strong et al in 1989, and since then, it has been reported as applied to a limited number of patients with BCS. According to the data reported in the publicly available literature, the first LDLT performed in a pediatric patient with BCS was attempted by Nezakatgoo et al^[12]^ in 1999. That same year, Haberal et al^[13]^ performed a heterotopic auxiliary left lobe LDLT in a 17-year-old female with BCS.

Akamatsu et al^[2]^ reported that only 32 LDLT procedures were performed in patients with BCS between 1999 and 2013. Those authors explained that the articles on this topic were completely confined to case reports (17 case reports and 3 case series). According to the registry data of the Japanese Liver Transplantation Society (JLTS), a total of 44 patients with BCS underwent LDLT between 1989 and December 2014, with survival rates of 60%, 85.4%, 82.4%, 82.4%, 72.1%, and 72.1% at 1, 3, 5, 10, 15, and 20 years, respectively.^[14]^ Of those 44 patients reported by the JLTS, 19 were also reported in the study by Akamatsu et al.^[2]^ Thus, we can conclude that a total of 57 patients with BCS underwent LDLT between 1989 and December 2014. Despite the small number of reported cases, the survival rates provided by these studies are quite satisfactory. However, the reported data from the JLTS leaves some questions that remain to be answered. First, what percentage of the higher survival rates account for cases of disease-free survival (or are they cumulative survival rates)? Second, what surgical technique was most frequently used in the reported cases (i.e., in more than a half of the reported cases) and what were the postoperative recurrence rates?

Almost all articles related to the use of cadaveric liver grafts in BCS were published by authors from Western countries, such as Europe and the United States, where the organ pool is largely based upon cadaveric donors. In DDLT, the type and localization of VCI do not present any challenges to the surgical technique. Instead, for cases undergoing DDLT, vascular continuity can be established by creating an anastomosis between the donor VCI and the recipient VCI following resection of thrombosed and/or fibrotic VCI.^[^2 3^]^ Thus, in countries where DDLT is very widely used, transplant surgeons do not avoid performing LT as a treatment of BCS, as is evidenced by the higher DDLT rates for BCS in those countries. In contrast, all of the studies related to LDLT in BCS were published from Asian countries, wherein the organ pool is largely based upon living donors. There is no possibility of using a donor's VCI in LDLT. Therefore, novel techniques aimed at eliminating stenosis or obstruction in the recipient VCI should be developed and a suitable orifice should be established for the implantation of the graft hepatic vein.^[3]^ To our knowledge, ∼70 patients worldwide with BCS underwent LDLT between 1989 and December 2015.^[^2 15^]^ This low number is most likely explained by the well-recognized technical difficulties of the implantation procedure for a graft obtained from living liver donors, and the related reservations of surgeons to perform such a procedure (ranging, for example, from risk considerations to an otherwise healthy individual who chooses to provide donation, to the inherent small experience level related to a procedure that is so rarely undertaken). Such technical difficulties become more apparent in BCS, where VCI is thrombosed in a significant proportion of patients.

The largest case series involving living liver donors in BCS reported to date contained 10 cases and was published by Kalayoglu et al.^[15]^ However, these authors reported that they exclusively performed reconstruction of the liver graft, without use of any cavoplasty technique for the VCI. It would be expected, therefore, that VCI deformation, webbing, or thrombosis occurred in none of the 10 cases. The second largest study published in the literature is the 9-case series reported by Yamada et al.^[3]^ In our study, presented herein, one of the most notable features is that it is now the largest and the most comprehensive study in the literature. Another important feature is the various reconstruction models used that had been designed involving both the venous drainage of the graft obtained from the living donor and the VCI of the recipient. Hence, we are of the opinion that the data from our study will help to guide surgeons or centers otherwise have reservations about the use of LDLT in BCS. The technical details of the use of living liver donors in BCS are summarized below to promote this benefit.

The most important factors underlying the choice of venous reconstruction technique to be used in patients with BCS are: whether VCI stenosis is complete, whether thrombus is extended to the right atrium, and whether the hemiazygos venous system if actively functional.^[3]^ Although no marked pressure gradient is created around the partial or complete stenotic segment in the VCI of patients with a well-developed venous system, a marked pressure gradient is frequently created around the partial stenotic segment in the VCI when the hemiazygos venous system is underdeveloped.^[3]^ In cases with no pressure gradient, an anastomosis can be created between the graft hepatic vein and intact suprahepatic VCI or right atrium after resection of the retrohepatic VCI is performed at the suprarenal level.^[3]^ Kazimi et al^[16]^ reported that they resected fibrotic and thrombosed VCI until the right atrium was reached and that they created an unproblematic anastomosis between the graft hepatic vein and right atrium. In cases where dissection around the retrohepatic VCI is difficult, an anastomosis can be created between the graft hepatic vein and intact suprahepatic VCI, without requirement of resection of the occluded VCI. Kubo et al^[17]^ reported that they were able to form a horizontal orifice on the anterior surface of the suprahepatic VCI to match the graft vein, without requirement of resection of a severely stenotic retrohepatic VCI. In the study presented herein, a patient underwent resection of the retrohepatic VCI to address the fact that it had become a fibrous band. Since the collateral system was well developed in that patient, an unproblematic end-to-end anastomosis was created between the graft hepatic vein and the suprahepatic VCI.

In cases with an existing pressure gradient, various reconstruction techniques for VCI may be designed to avoid some complications, such as lower extremity edema, ascites, and renal dysfunction. First, the deformed retrohepatic VCI should be resected after applying a cross-clamp to the intact supra and infrahepatic VCI segments, and venous continuity should be established with grafts of synthetic materials (Gore-Tex, polytetrafluoroethylene [PTFE], silver-coated polyethylene terephthalate [Dacron])^[18]^ or allogeneic materials (aorta, iliac artery/vein, VCI, peritoneal fascial graft, bovine pericardium).^[^5 19–22^]^ In the current study, we used various allogeneic vascular grafts for VCI reconstruction in 6 of the patients.

Second, in cases with intraluminal thrombus in VCI, a part of the anterior wall which includes the hepatic vein stump should be excised and thrombectomy should be performed after the native liver is explanted. After thrombectomy, an anastomosis should be created between the graft hepatic vein (previously prepared on the backtable) and the orifice formed in the VCI. Soyama et al^[23]^ reported that they longitudinally opened the VCI and then performed a massive thrombectomy and anastomosis. In our opinion, for cases of BCS with a partly deformed or thrombosed VCI, performing thrombectomy alone may not be sufficient; moreover, such cases are typically characterized by an increased risk of recurrence. In a significant percentage of our cases, presented herein, we performed the thrombectomy first; however, we did not confine our surgical approach to thrombectomy alone and we proceeded with additional reconstruction techniques.

Third, some cavoplasty techniques that are available to widen the lumen of a stenotic VCI may be of use in cases with severe mural thickening. Yamada et al^[3]^ dissected the dense tissues around the vein cranially until the intact vein wall was reached, and then resected the thickened anterior surface of the thrombotic and stenotic VCI until the intact borders were reached. A new and wide orifice was obtained by wrapping the vein graft around the defect that had developed on the VCI; the authors designated that procedure as ‘venous patch plasty’. Similarly, we performed the procedure (in 32 of 39 patients reported herein) by dissecting the fibrous and dense tissues around the VCI, followed by resecting of the anterior and right/left lateral walls of the stenotic VCI cranially and caudally until the intact vein wall was reached. Then, we created an anastomosis between the VCI and the graft hepatic vein, which had been formed as a circumferential fence on the backtable. Although Yamada et al^[3]^ had wrapped the venous graft around the defect that had formed in the native VCI in order to create the new orifice, we had wrapped a vein graft around the graft hepatic vein and formed a wide orifice. In our study population, 5.1% of the patients developed recurrences; this number was much lower than that in the study of Yamada et al.^[3]^, which reported a recurrence rate of 40%.

Thrombosis, fibrosis, or mural thickening in the VCI may extend to the right atrium. In such cases, carrying out massive thrombectomy or reconstruction in subdiaphragmatic VCI will usually be insufficient. In those cases with well-developed collateral circulation, an anastomosis may be created between the graft hepatic vein and the right atrium (by use of allogeneic or synthetic vascular grafts) after resection of the VCI segment that remains proximal to the renal veins. Choi et al^[19]^ reported their success in creating adequate drainage in a case with complete obliteration of retrohepatic vein by placing a PTFE vascular graft between that graft hepatic vein and the right atrium. Akamatsu et al^[2]^ a similar success by use of a cryopreserved VCI graft placed between the graft hepatic vein and right atrium.

Reported rates of BCS recurrence following DDLT range from 0% to 33.3%. This condition is closely related to 2 factors: anastomotic stenosis associated with the surgical technique and tendency for hypercoagulability associated with the underlying disease.^[3]^ To avoid thrombosis, the INR should be kept between 1.5 and 2, particularly in cases of BCS due to hematological disorders. We administered heparin at the early postoperative period after LDLT for BCS, which was followed by a switch to warfarin-acetylsalicylic acid combination therapy that was continued for 6 months. A primary limitation of our study involves this therapy; a detailed search for etiology of all BCS cases treated in our study was not performed and analyses for JAK2, protein C and protein S were carried out for only 10 of the total 39 patients, with only 2 of those patients showing abnormal results and receiving long-term warfarin therapy.

The lack of a detailed search for the etiology of all BCS cases in this study also precluded our ability to recommend an appropriate algorithm for postoperative follow-up. Almost all cases referred to our clinic for BCS have already participated in consultation with the hematology department, which—for the past 2 years—has also collaborated in these patients’ management.

In conclusion, despite the utilization of many medical, radiological, and surgical shunting interventions for the treatment of BCS, LT remains the most frequently applied and successful treatment modality. Although the case reports from countries where DDLT is performed routinely have not reported any serious technical problems, countries where LDLT is more routinely carried out still struggle with this issue. The limited number of case reports of LDLT performed in BCS is the most important indicator of such a concern. Herein, we aimed to report the largest and most detailed study of LDLT for BCS to date worldwide. In our opinion, LDLT may be applied successfully and the recurrence rates can be lowered to acceptable limits in BCS. Any of the techniques reported here can be readily applied. The basic criteria to achieve successful outcomes in these cases include determination of the actual underlying cause, experience of a center in the surgical techniques to be applied, and quality of the postoperative patient management.

## Acknowledgments

The authors thank Prof Dr Sezai Yilmaz, Director of Liver Transplant Institute, for the support to us in the design of this study. They also thank Prof Dr Sezai Yilmaz for the efforts in the establishment of first Liver Transplant Institute in Turkey.
